# Change of temperature field around different drainage structures in cold region tunnel based on model testing

**DOI:** 10.1038/s41598-023-41175-5

**Published:** 2023-08-26

**Authors:** Jinhuan Zhu, Xuelan Zhang, Lanjun Liu, Lizhen Tan, Lulu Liu

**Affiliations:** 1 School of Civil Engineering, Shangqiu Institute of Technology, Shangqiu, 476000 China; 2 School of Highway, Chang’an University, Xi’an, 710064 China; 3 State Key Laboratory for Geomechanics and Deep Underground Engineering, China University of Mining and Technology, Xuzhou, 221116 China

**Keywords:** Environmental sciences, Natural hazards, Solid Earth sciences

## Abstract

Improper layout of drainage structures and inadequate insulation measures in cold tunnels can result in varying degrees of frost formation during operation. This study focuses on the Hongtoushan highway tunnel as an example, where the distribution characteristics of the temperature field around the lower drainage structure under different arrangements are investigated through indoor model testing. The results indicate that there is a significant hysteresis phenomenon in temperature changes across the cross-section as the burial depth increases. With an increase in the burial depth of the surrounding rock, the hysteresis time of temperature changes gradually elongates. The temperature variation pattern can be approximated by a cubic polynomial. In the vertical section, as the tunnel depth increases, the temperature of the surrounding rock in the lower part of the tunnel gradually rises while the amplitude of temperature change diminishes. The temperature near the centerline is relatively lower compared to the sides, where the temperature gradually increases moving away from the centerline.

## Introduction

Due to the large-scale development of western infrastructure, tunnels built in local areas are becoming insufficient to meet the regional demands. As a result, tunnels are being constructed at higher latitudes, altitudes, and in colder regions with extremely harsh natural conditions^1–3^. Building tunnels in such areas brings about more complex climatic, geological, mechanical, and technical challenges^4^. Moreover, it is necessary to consider the impact of severe cold weather on the frost resistance of portal structures and drainage facilities, as well as the safety of tunnels during operation. In particular, tunnel construction in cold regions must take into account a series of cold-related problems, such as the freezing of tunnel drainage and frost heave of drainage pipelines, which are significant factors that cause tunnel diseases^5^. These issues must be addressed to ensure the safe and efficient operation of tunnels in such regions.

Common issues in cold tunnels include water leakage, lining cracking, and freeze–thaw damage^6,7^. Among these, water seepage and freeze–thaw can have a significant impact. In addition to considering the construction characteristics of regular tunnels, cold tunnels face the additional challenge of frost damage to drainage facilities. In recent decades, China has constructed many cold tunnels. Through on-site research, it has been found that due to the extremely harsh weather conditions and unreasonable placement of drainage facilities, about 80% of alpine cold tunnels have some degree of frost damage. This not only affects their normal operation but also causes major traffic accidents, posing significant difficulties for later maintenance and prevention^8–10^. For example, the Qidaoliang Tunnel located in Gansu Province and the Dabanshan Tunnel located in Qinghai Province in China have experienced various diseases such as severe water leakage, ice hanging, and lining cracking during the severe cold season. The freezing of the drainage ditch makes it problematic to drain the surrounding rock and groundwater behind the tunnel lining, causing the surrounding rock to freeze and heave, seriously affecting the normal use of the tunnel and consuming a large amount of assets^11,12^. According to recent statistical surveys in Japan, nearly 30% of railway tunnels have a certain degree of frost damage. In the Hokkaido region of Japan, the frost damage problem of the waterproof and drainage structures of large highway tunnels seriously affects the normal use of tunnels^13^. Therefore, in the process of tunnel construction in alpine cold regions, solving the problem of tunnel lining insulation and frost prevention, as well as setting up a reasonable waterproof and drainage system, are critical to preventing tunnel frost damage.

Traditional techniques for blocking and draining leakage water and treating lining cracks are gradually unable to meet the current needs, and new research on drainage structures is needed^10–14^. Kim et al.^15^ studied the causes of water seepage in alpine cold tunnels by developing new tunnel drainage structures. Liu et al.^16^ introduced the basic principles, ideas, and specific measures for the treatment of diseases such as water leakage, insulation, and lining reinforcement in the Dabanshan cold Tunnel by analyzing the causes of the diseases of tunnels. To reduce the risk of blockage in the tunnel drainage system, Liu et al.^17^ determined the distribution of defects in highway tunnels through on-site investigation, indoor experiments, and literature analysis, and proposed an optimization method for tunnel drainage structure. Ji et al.^18^ proposed that the circular drainage pipe should be directly connected to the central drainage pipe, and a strip insulation layer should be installed in areas where the surrounding rock is rich in water and the middle and lower parts of the circular drainage pipe are prone to freezing. Connecting the circular drainage pipe to the central drainage pipe in the cold channel is beneficial for tunnel insulation. In order to strictly waterproof and improve the tunnel drainage system, Lu et al.^19^ proposed a plan for the insulation layer of the decorative strip in front of the tunnel circular drainage pipe. The circular drainage pipe is directly connected to the central drainage pipe, and the longitudinal drainage pipe is connected to the circular drainage pipe through a tee^19^. Luo et al.^20^ proposed anti-freezing measures using central trenches, deep central trenches, thermal protective layers, and drainage holes to address the varying degrees of freezing damage in tunnels. The structural form of using composite lining and setting waterproof layers between the lining is not only beneficial for waterproofing and drainage, but also for frost prevention. The research results of the above scholars fully prove that waterproofing in cold tunnels is the foundation, drainage is the core, and frost prevention is the key.

This article focuses on the Hongtu Mountain Tunnel located on the S308 line in Qinghai Province. In order to study the impact of insulation conditions on the temperature field distribution around the tunnel drainage structure, a scale model test was conducted. The test results obtained from both the cross-section and vertical section of the tunnel, taking into account the depth of surrounding rock and tunnel depth, were analyzed to understand the distribution characteristics of the temperature field. The ultimate goal of this research is to mitigate tunnel-related issues caused by inadequate drainage in cold tunnels, while also providing a valuable insights for the construction and safe operation of tunnels.

## Test scheme

### Site conditions

The project relies on the Hongtushan Tunnel in Qinghai Province, with complex and varied terrain and landforms, with an elevation of approximately 4000–4800 m. The Hongtushan Tunnel is a single hole bidirectional driving tunnel with a net width of 13.0 m and a net height of 5.0 m. The entrance road surface elevation of the tunnel is 4280 m, and the exit road surface elevation is 4350 m, The total length of the tunnel is 3170 m. The designed road surface elevation is 4280.83–4352.90 m, the designed longitudinal slope is 2.4%. The axis direction is 261° and the maximum burial depth of the tunnel is 277 m. The Red Soil Mountain Tunnel project is situated in the heartland of an inland plateau, boasting high altitudes, relatively low temperatures, abundant sunlight, strong solar radiation, and a typical plateau continental climate characterized by concurrent rainy and hot periods. The tunnel's location experiences prolonged winters and brief summers, with blurred seasonal transitions and substantial temperature variations between day and night. The annual average temperature stands at 0.4 °C, with an average highest temperature of 11.7 °C and an average lowest temperature of − 3.2 °C. Extreme temperatures can soar up to 28.7 °C at their peak and plummet to − 27.6 °C at their coldest. The air is thin, and there exists no definitive frost-free period. The maximum freezing depth measures approximately 2.98 m. The hydrogeological conditions in the tunnel site area are exceptionally intricate, primarily attributed to the widespread occurrence of seasonal frozen soil. This characteristic adds a distinctive aspect to the area. Consequently, considering factors such as the distribution patterns of underground water along the route, the region can be classified into three distinct categories: the upper water layer situated above the frozen layer in the seasonal frozen soil zone, the lower water layer below the frozen layer, and the groundwater within the thawed zone.

### Model experiment design

To satisfy the site requirements, it is necessary for the tunnel model to conform to geometric similarity, time and temperature similarity, thermal conductivity similarity, and specific heat capacity similarity. The accuracy of the specific similarity is based on the research method proposed by Lai et al.^21^.

There are three major similarity theorems that should be followed in model testing: the theorem of similarity, the π theorem, and the inverse similarity theorem. The tunnel model and the prototype should satisfy the following similarity requirements: geometric similarity, boundary condition similarity, and thermodynamic parameter similarity.

1. Geometric similarity: The geometric dimensions of the model test should be scaled down proportionally according to the actual dimensions of the tunnel. The length similarity ratio is given by:
  1 $$ C_{l} = \frac{{l_{p} }}{{l_{m} }} $$

From the length similarity constant, the area similarity ratio and volume similarity ratio can be derived as:

2 $$ C_{A} = \frac{{A_{p} }}{{A_{m} }} = \frac{{l_{p}^{2} }}{{l_{m}^{2} }} = C_{l}^{2} $$

3 $$ C_{V} = \frac{{V_{p} }}{{V_{m} }} = \frac{{l_{p}^{3} }}{{l_{m}^{3} }} = C_{l}^{3} $$

Here, *C* represents a constant, *l* represents size, *p* represents the prototype, and *m* represents the model.

2. The boundary conditions in tunnel model tests may vary with time and temperature, and the similarity of the boundary conditions in terms of time and temperature should also be maintained.
3. The thermal conductivity and Specific heat capacity of the materials selected for the tunnel model test, as well as the thermal insulation and antifreeze materials, should be similar to the prototype.

Considering factors such as experimental operability, the geometric reduction ratio of the tunnel cross-section was established as 1:24, despite the total depth of the tunnel prototype being 3020 m. This was determined as the optimal ratio for the scaled model test. For structures with a relatively large slenderness within the tunnel, a variable rate model should be utilized if a scaled model test is conducted on the entire structure. In this study, the variable rate in the depth direction of the tunnel model was determined to be 41.94, and the depth of the tunnel model was set at 3 m, the inner diameter of the tunnel is 30 cm, the thickness of the insulation layer is 3 cm.

The burial depth of the central drainage ditch in the original tunnel is 2.9 m, while the cold relief tunnel has a burial depth of 4.5 m. Due to limitations in the test site conditions and testing operability, a geometric reduction ratio of 1:24 was determined for the burial depth of the drainage structure. Consequently, the burial depth of the central drainage ditch was determined to be 12 cm, while the burial depth of the cold relief tunnel was established to be 19 cm. The pipe diameter of the central drainage ditch was also determined to be 2.0 cm.

### Test material ratio

The final mix ratio of lining concrete is determined as: water: cement: sand: stone = 0.38: 1: 1.11: 2.72, with a thermal conductivity of 2.410 W/m K, which is approximately equivalent to the model standard C25 concrete thermal conductivity of 2.461 W/m K.

The mix ratio of surrounding rock materials are: water: sand: lime: stone: soil = 0.35: 1.5: 0.2: 0.6: 2.0, and the thermal conductivity is taken as the average value of 2.64 W/m K for different types of rocks used. The parameters of surrounding rock materials are shown in Table 1.

**Table 1:** Surrounding rock materials’ physical and mechanical parameters.

Group	Material								
	Water (Kg)	Cement (Kg)	Lime (Kg)	Sand (Kg)	Stones (Kg)	Granite particles (Kg)	Iron powder(Kg)	Early strength agent (Kg)	Thermal conductivity (W/m K)
Group 1	2.3	3.8		8.8	1.14	1	0.2	0.03	2.348
Group 2	2.3	3.8		10	1.14	2	0.4	0.03	1.492
Group 3	1.5	2.5		5	5	0	0	0	1.302
Group 4	0.38	1		1.11	1.36	0	0	0	1.526
Group 5	0.375		0.375	1.5	0.625	Soil 1.5			1.452
Group 6	0.375		0.275	2.0	0.625	Soil 1.5			1.551

### Test preparation

**Figure 1:**
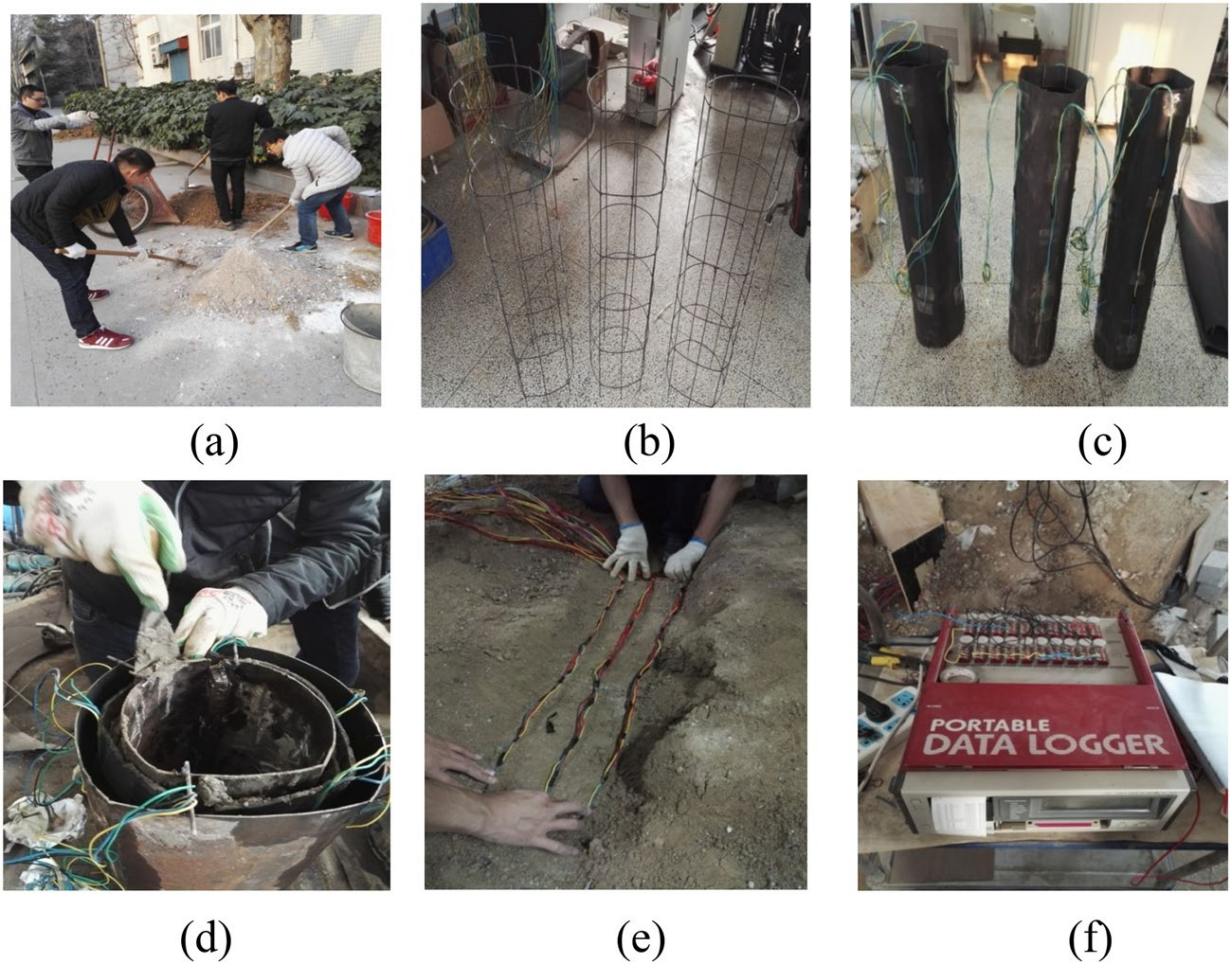
Prefabrication of model device. (**a**) Mixing of surrounding rock materials; (**b**) model tunnel steel wire mesh layout; (**c**) insulation layer and sensor layout; (**d**) concrete pouring; (**e**) thermometer embedding; (**f**) data acquisition.

The thermal conductivity of the surrounding rock material of the tunnel model is similar to that of the on-site tunnel surrounding rock. A reasonable material ratio is obtained through the ratio test, and then various materials are mixed evenly in proportion according to the ratio test results. After the model production is completed, the model is poured. In order to prevent the model hills from being too heavy, the concrete strength grade can be appropriately increased, and C30 grade concrete can be used. The experimental model preparation is shown in Fig. 1. The production of tunnel models is based on the research plan of Liu et al.^11^. Liu et al.^11^ refereed to the site conditions of Dabanshan Tunnel and the operability of model test, the indoor model tunnel was constructed by scale reduction, with the tunnel length of 3 m and internal and external diameters of 12.2 cm; 13.2 cm. The surrounding rock materials of the tunnel are prepared based on on-site conditions, using 7.5% water, 32.2% sand, 4.3% lime, 12.9% level, and 43.0% soil^11^.

**Figure 2:**
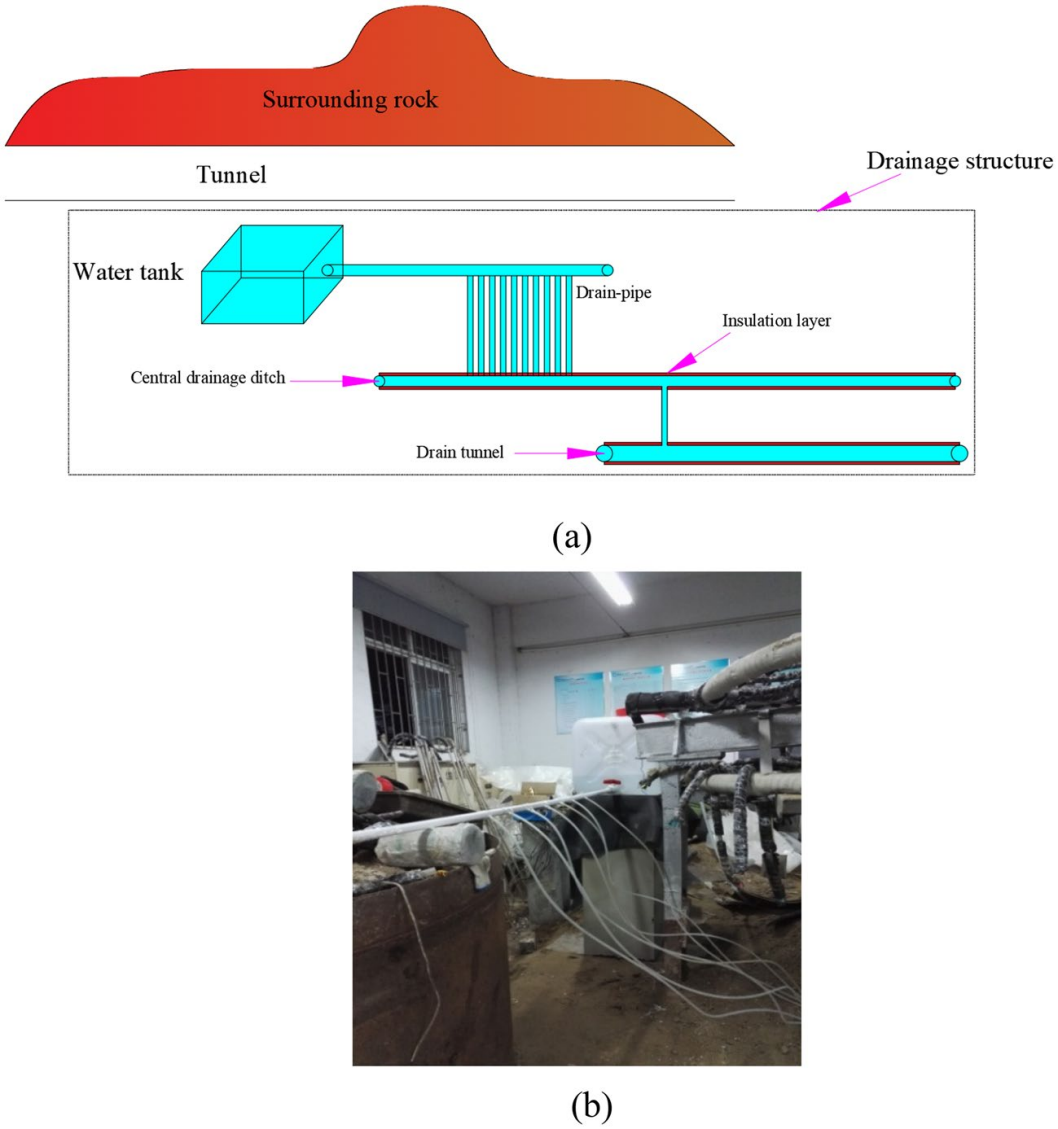
Layout of drainage system. (**a**) Schematic diagram of drainage structure; (**b**) drainage structure layout.

In the experiment, PVC pipes were utilized as both the central drainage ditch and the anti-cold drainage holes. The two were then connected using an 8 mm hose. A water tank is placed at a certain distance above the top of the tunnel, and the outlet of the water tank is connected to a PVC pipe. A drainage valve is set on the PVC pipe to control the water flow by adjusting the valve. Figure 2 shows the layout of the drainage system.

**Figure 3:**
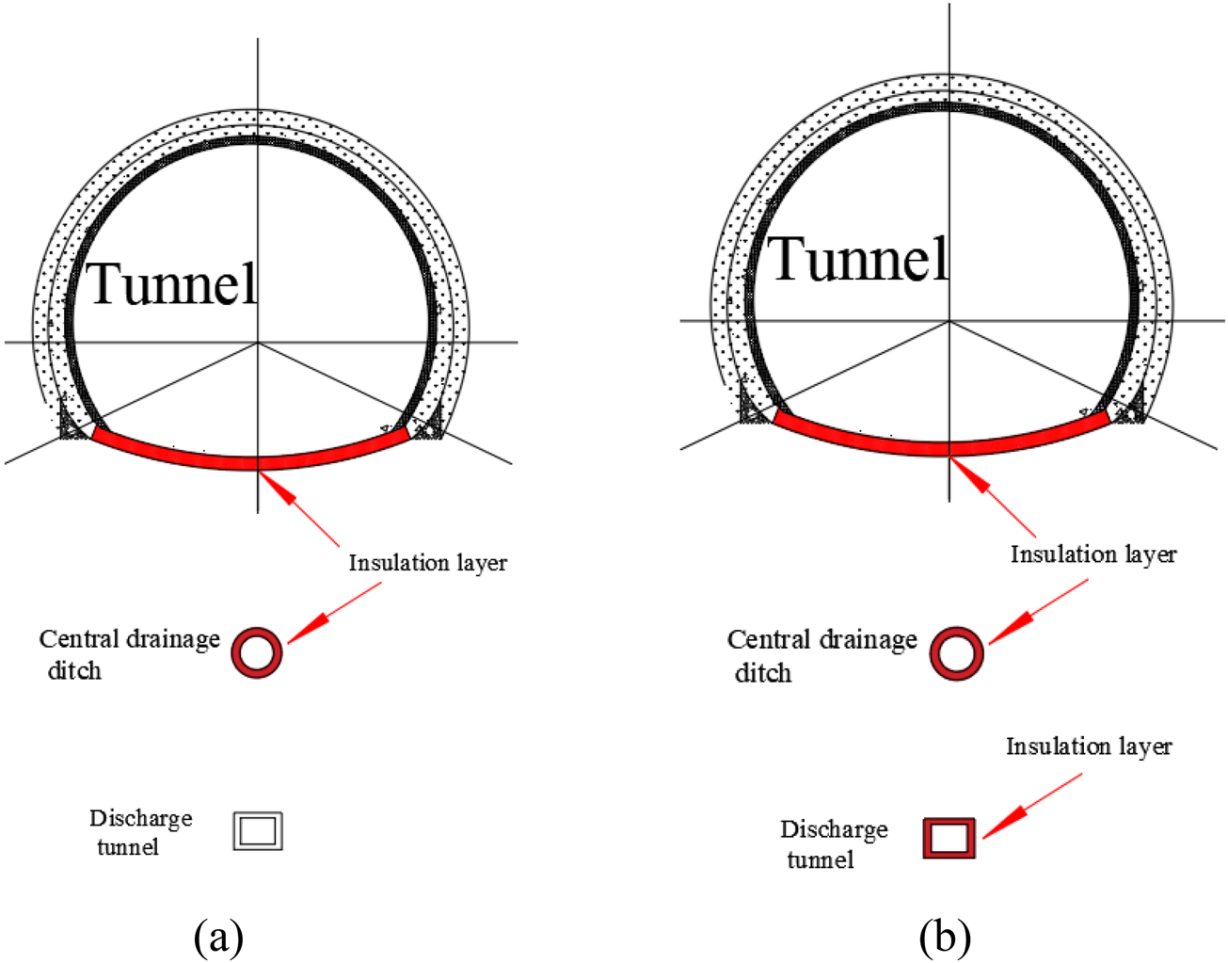
Thermal insulation scheme for tunnel drainage structure. (**a**) Scheme I; (**b**) scheme II.

Two drainage test schemes were conducted. Scheme I involved setting insulation conditions for the inverted arch and the central drainage ditch, while Scheme II involved setting insulation conditions for the inverted arch, central drainage ditch, and anti-cold drainage tunnel. Specific insulation conditions are detailed in Fig. 3.

**Figure 4:**
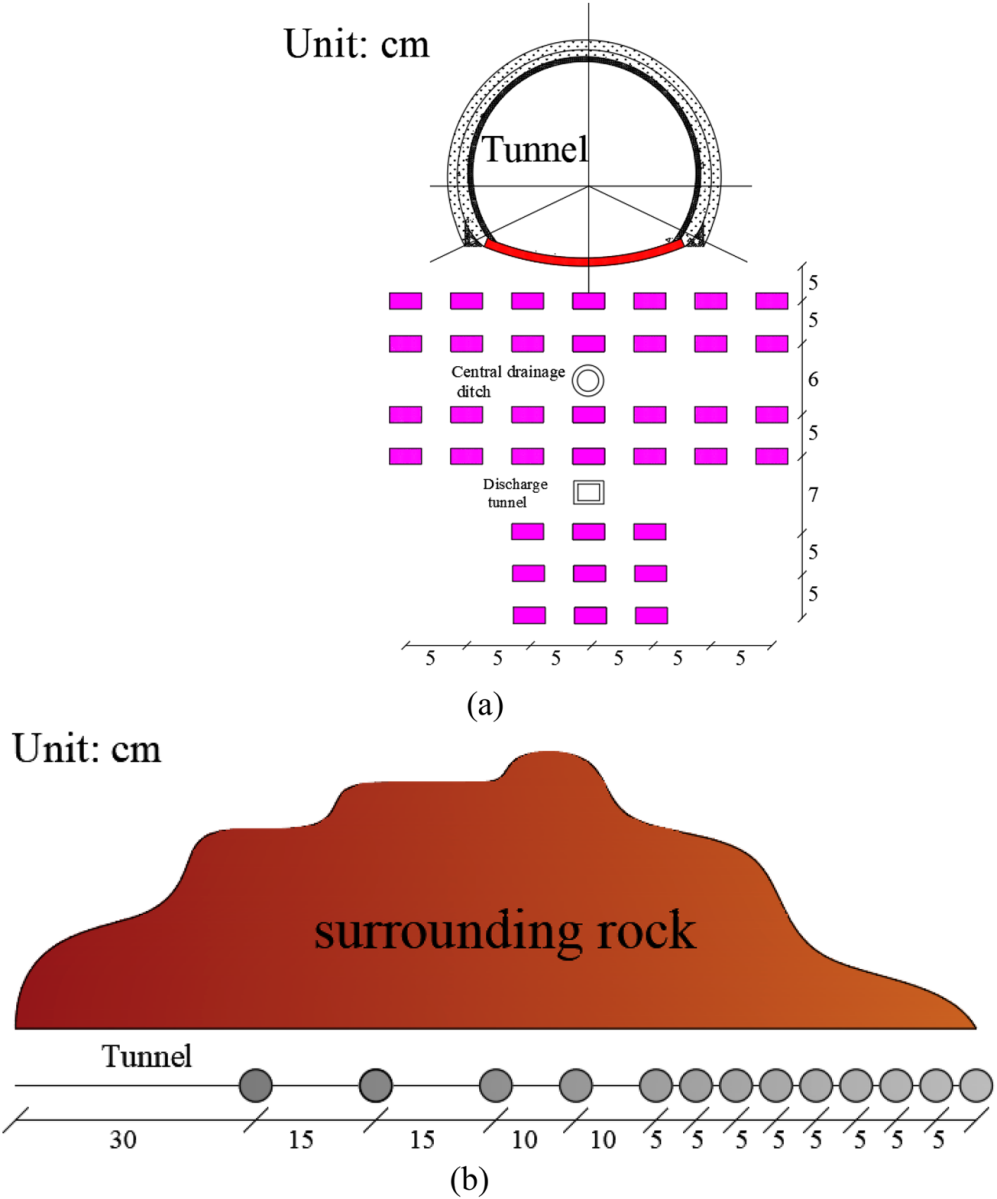
Layout diagram of temperature sensor. (**a**) Transverse section; (**b**) longitudinal section.

After the completion of the temperature sensor and drainage structure layout, the model tunnel is placed and the model hill is stacked. In order to better simulate the freezing conditions of the tunnel, the water flow rate and the distribution of temperature field around the drainage structure, four freezing pipes are installed on both sides of the tunnel surrounding rock hill to simulate the freezing effect of cold air outside the tunnel on the tunnel. A temperature sensor schematic indicating deployment at the transverse and longitudinal sections of the tunnel is illustrated in Fig. 4.

At the transverse section, assuming the distance from the central drainage ditch to the inverted arch is unit 1, the burial depths of the measuring points at different depths are 5/13 x, 10/13 x, 16/13 x, 21/13 x, 28/13 x, 33/13 x, and 38/13 x, respectively.

The longitudinal section assumes that the total depth of the model tunnel is defined as 1. Taking the 5 cm depth of the tunnel as an example, it is represented as 1/60 of the total depth of the tunnel. The other section positions are consistent with this representation method.

### Consent to publish

All subjects and their legal guardians are aware of and agree to use it for publishing identification information and images in online open access publications.

## Results and analysis

### Analysis of drainage test results under insulation conditions of tunnel invert and central drainage ditch

To assess the effectiveness of the tunnel drainage structure, various factors were studied. This includes whether the drainage pipeline freezes under low-temperature conditions and if such freezing adversely affects the normal use of the drainage system. Additionally, whether the insulation measures set up in the drainage structure offer significant insulation effects was also studied. As such, insulation boards were placed to insulate both the inverted arch and central drainage ditch, and freezing tests were then conducted. Through this, temperature data was collected at each measuring point, allowing for determination of the temperature distribution characteristics of the tunnel's drainage structure at various transverse and longitudinal section positions.

#### Temperature distribution characteristics around drainage structure of tunnel transverse section

**Figure 5:**
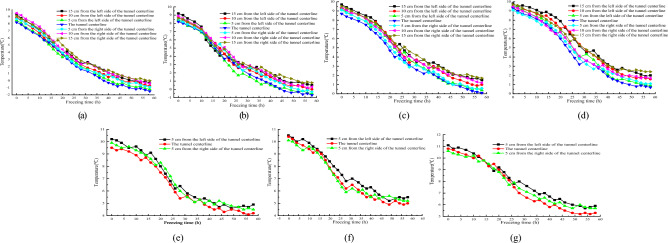
Relationship between transverse sectional temperature and freezing time at the tunnel portal location. (**a**) 5/13 x; (**b**) 10/13 x; (**c**) 16/13 x; (**d**) 21/13 x; (**e**) 28/13 x; (**f**) 33/13 x; (**g**) 38/13 x.

Figure 5 shows the variation curve of surrounding rock temperature with freezing time at different positions on the transverse section at a distance of 1/60 from the tunnel opening outside the tunnel.

Based on the information presented in Fig. 5, it can be observed that at shallower burial depths, as the freezing test commences, the temperature gradually decreases at a rate of roughly 0.25–0.35 °C/h. However, as the burial depth of the lower surrounding rock increases, a hysteresis phenomenon is observed in the temperature change. At the onset of freezing, the temperature drop rate slows down to about 0.1 °C/h, and the temperature change curve becomes relatively flat. The lag time of temperature change varies with different burial depths, with longer lag time observed at deeper burial depths, The farther away from the bottom of the tunnel invert, the less likely the cold air inside the tunnel is to affect the temperature changes of the surrounding rock. Therefore, there is a significant hysteresis period in the temperature under the deep surrounding rock. For instance, at 10/13 times the burial depth of the central drainage ditch, the lag time of temperature change is 10 h. In contrast, at 38/13 times the burial depth of the same drainage ditch, the lag time increases to 22 h. During the lag time period, the temperature at each measuring point gradually decreases with an average decrease rate of around 0.1 °C/h. When the freezing time surpasses the lag time, the rate of temperature change rapidly increases, resulting in a sharp temperature drop at a rate of about 0.3 °C/h, which is approximately 2–3 times the temperature drop rate during the lag time period. Once the temperature has dropped to a certain extent, the exchange of cold and hot air approaches equilibrium, leading to a significant reduction in the rate of temperature drop, ultimately stabilizing the temperature.

The lag time of temperature changes at different burial depths on the transverse section of 1/60 tunnel depth and 1/60 tunnel depth outside the tunnel are shown in Table 2 and Fig. 5.

**Table 2:** Lag time at different burial depth locations.

Depth	(5/13) x	(10/13) x	(16/13) x	(21/13) x	(28/13) x	(33/13) x	(38/13) x
Latency /h	0	10	16	16	18	20	22

Note: (a) x represents a multiple of the distance from the bottom of the inverted arch.

**Figure 6:**
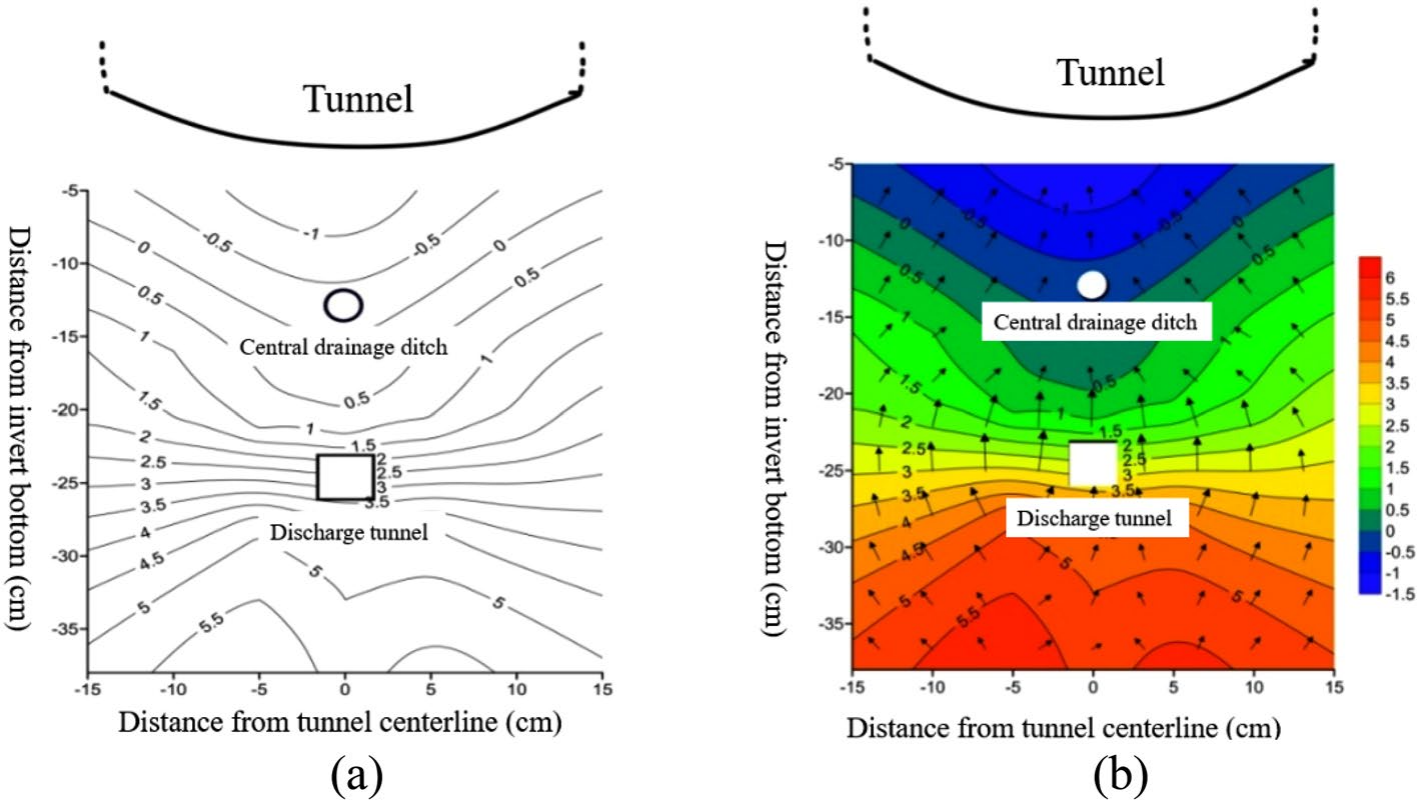
Temperature changes at different locations on the tunnel transverse section. (**a**) Temperature contour map of tunnel transverse; (**b**) temperature vector map of tunnel transverse.

Figure 6 shows the variation of surrounding rock temperature at different positions on the transverse section at a distance of 1/60 from the tunnel opening outside the tunnel.

From Fig. 6, it can be seen that at the same burial depth, the temperature value at the center line of the tunnel is the lowest. As we move towards the left and right sides, the temperature value gradually increases, reaching its peak at a distance of 15 cm from the center line. The maximum temperature difference between the center line of the tunnel and each position on the left and right sides is approximately 1.7 °C. This temperature difference corresponds to burial depths of 5/13, 10/13 times the central drainage ditch, and 16/13 times the burial depth of the same drainage ditch. Additionally, as the burial depth of the surrounding rock increases, the temperature values of each measuring point weaken due to atmospheric temperature changes and the influence of freezing environment temperature. Shallow buried surrounding rock is highly affected by the exchange of cold and hot air and experiences a significant reduction in temperature, whereas deeper buried surrounding rock tends towards positive temperature and the minimum internal temperature gradually increases. A negative temperature distribution area is observed within the range of 16/13 times the burial depth of the central drainage ditch to the depth of the tunnel invert, with temperatures ranging from approximately − 0.1 to − 0.5 °C and a minimum temperature of − 0.5 °C. Meanwhile, a positive temperature distribution area is detected around the anti-cold drainage tunnel, with a temperature range of 1.1–2.5 °C, a minimum temperature of 1.1 °C, and a maximum temperature difference of approximately 1.4 °C. The temperature variation curve in Fig. 6 is fitted using data-fitting techniques and is represented by a cubic polynomial equation. The parameters of the fitting equation can be found in Table 3.

**Table 3:** Parameters of fitting curve.

Parameter		Place						
		(5/13) x	(10/13) x	(16/13) x	(21/13) x	(28/13) x	(33/13) x	(38/13) x
Outside the tunnel	C	9.52754	8.56168	9.53867	9.67786	9.64619	10.52962	11.17068
	*B*_1_	− 0.30538	− 0.19533	− 0.13263	0.03405	− 0.04838	− 0.05377	− 0.07698
	*B*_2_	0.00237	− 0.00202	− 0.0034	− 0.0094	− 0.0046	− 0.00364	− 0.0024
	*B*_3_	− 3.51E−7	4.65 E−5	5.49E−5	1.13E−4	6.72 E−5	5.32 E−5	3.70 E−5
	$R^{2}$	0.99439	0.99286	0.98335	0.99031	0.98782	0.98779	0.99177
Inside the tunnel	C	7.73883	8.91174	8.44257	8.79824	11.42223	11.13043	11.04834
	*B*_1_	− 0.22961	− 0.09989	− 0.10007	0.01219	− 0.03207	− 0.03092	0.00742
	*B*_2_	− 0.00042	− 0.00662	− 0.00458	− 0.00876	− 0.00593	− 0.00522	− 0.00667
	*B*_3_	2.94E−5	9.59 E−5	7.11 E−5	1.11 E−4	7.52 E−5	6.82 E−5	8.15 E−5
	$R^{2}$	0.99511	0.99674	0.98592	0.99148	0.99459	0.99189	0.99494

Note: (a) x represents a multiple of the distance from the bottom of the inverted arch.

From Table 3, it can be seen that the variance of the fitting equation is basically above 0.99, indicating a high degree of data fit. The variation of temperature with freezing time can be represented by a cubic polynomial.

To summarize, when the inverted arch and central drainage ditch are insulated, the temperature of various sections of the tunnel undergoes changes as the freezing time progresses. Shallow buried surrounding rock experiences a rapid decrease in temperature, which further decreases with an increase in freezing time during the early stages of freezing. As the burial depth increases, the temperature change at each measuring point exhibits a significant lag phenomenon, and this lag time tends to increase gradually with the burial depth of the surrounding rock^23^. The temperature variation curve with freezing time at each measuring point position follows an approximately cubic polynomial form, represented by:

4 $$ T = C + B_{1} t + B_{2} t^{2} + B_{3} t^{3} $$

Here, *T* represents temperature; C, B represent constant;* t* represents time.

At the same burial depth, the temperature near the centerline of the tunnel is relatively low, with an increasing temperature value as we move away from the centerline towards the left and right sides. It is worth noting that the temperature variation pattern at the depth of the tunnel differs from that observed in the outer section. Specifically, while the temperature at the center line of the tunnel is lower than that within the range of 10 cm on both sides, the temperature is the lowest at the 15 cm position on both sides of the center line. Moreover, the temperature of the surrounding rock gradually increases at the same location with an increase in burial depth, while the amplitude of temperature change tends to decrease gradually with the increase in burial depth.

#### Temperature distribution characteristics around tunnel axial drainage structure

**Figure 7:**
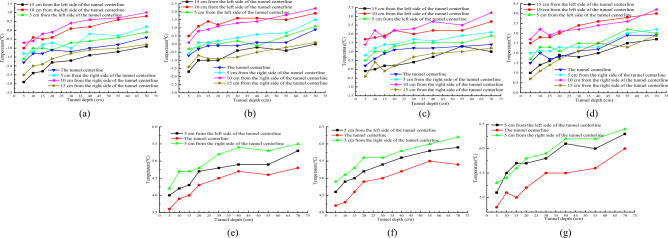
Longitudinal temperature variation curve at different positions in the tunnel. (**a**) 5/13 x; (**b**) 10/13 x; (**c**) 16/13 x; (**d**) 21/13 x; (**e**) 28/13 x; (**f**) 33/13 x; (**g**) 38/13 x.

**Figure 8:**
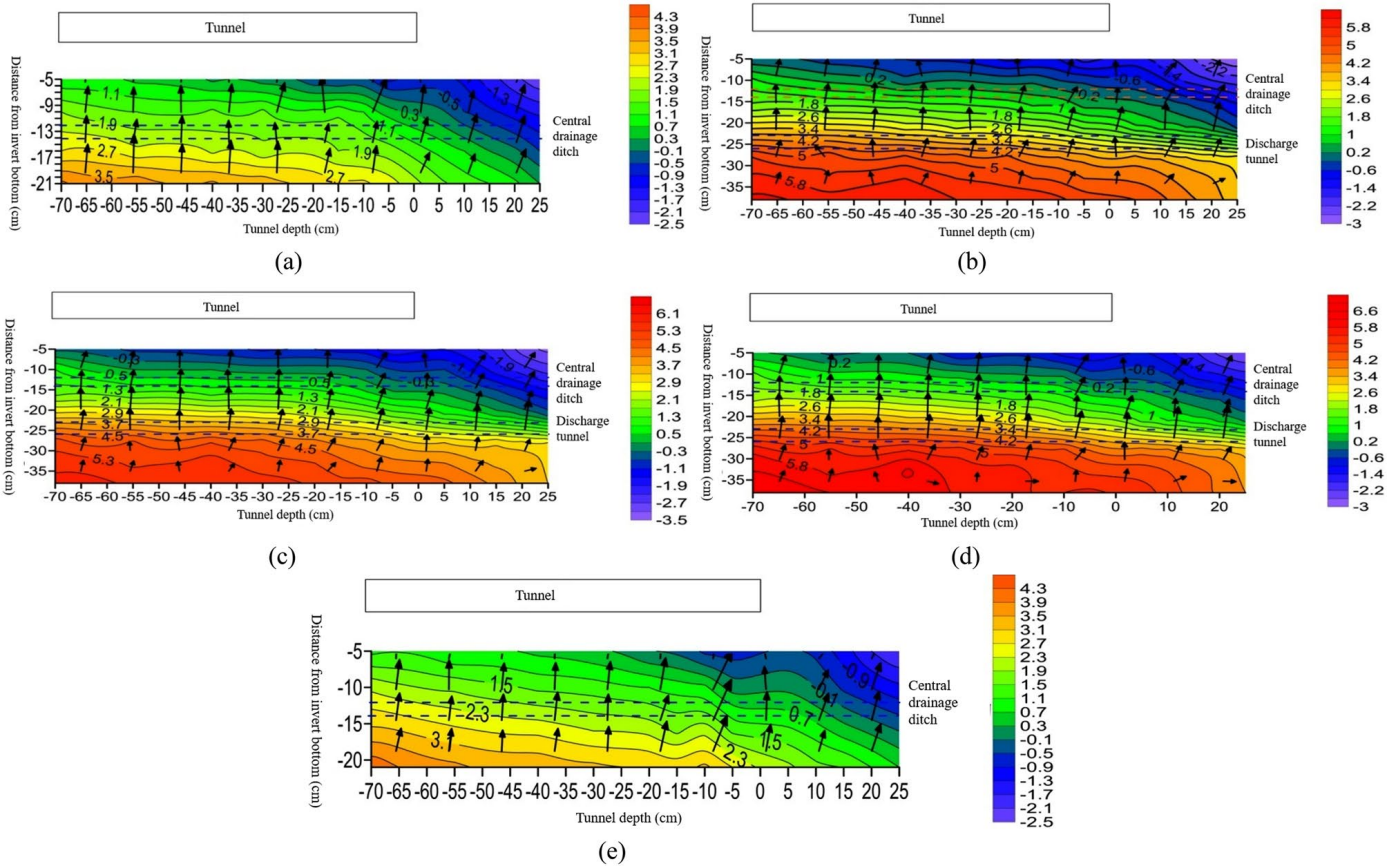
Vector diagram of longitudinal temperature changes at different locations of the tunnel. (**a**) Temperature variation vector map at 10 cm on the left side of the tunnel centerline; (**b**) temperature variation vector map at 5 cm on the left side of the tunnel centerline; (**c**) vector map of temperature changes at the centerline of the tunnel; (**d**) temperature variation vector map at 5 cm on the right side of the tunnel centerline; (**e**) temperature variation vector map at 10 cm on the right side of the tunnel centerline.

Figure 7a–g show the longitudinal temperature variation curves at different burial depths in the tunnel, Fig. 8a–e show the contour and vector maps of the longitudinal temperature variation at different positions in the tunnel.

From Fig. 8a and e, it is observable that the temperature changes along the axial direction at 10 cm positions on both sides of the tunnel centerline are nearly identical. From a depth of 7/30 of the tunnel to the position near the tunnel entrance, the temperature values at different burial depths below the tunnel arch decrease as the tunnel depth decreases. The temperature value is the lowest at 1/12 of the tunnel depth outside the tunnel entrance. Negative temperatures begin appearing around the central drainage ditch outside the tunnel, while positive temperatures occur within the depth range of the tunnel.

From Fig. 8b–d, the temperature variation along the axial direction at the centerline of the tunnel and its 5 cm positions on both sides is roughly shown as follows: from a depth of 7/30 of the tunnel to 1/12 tunnel depth outside the tunnel, the temperature decreases with the decreasing tunnel depth. The highest temperature at the same burial depth occurs at 7/30 tunnel depth, and the lowest temperature occurs at 1/12 tunnel depth outside the tunnel. There is a negative temperature distribution around the central drainage ditch at 5 cm on both sides of the tunnel centerline, but there are slight variations in the negative temperature distribution areas. There is no negative temperature around the anti-cold drainage tunnel, and all measuring points are positive. The temperature change pattern indicates that as the depth decreases, the temperature gradually decreases.

In summary, under the insulation conditions of the tunnel arch and central drainage ditch, negative temperature still exists within a specific range around the central drainage ditch. The temperature value gradually increases with increasing tunnel depth, and the amplitude of temperature increase gradually decreases. Due to the limited number of freezing pipes arranged outside the tunnel, the temperature values on the left and right sides of the tunnel centerline are relatively low (Li et al.^12^).

### Analysis of drainage test results under insulation conditions for inverted arches, central drainage ditches, and anti-cold drainage tunnels

#### Temperature distribution characteristics around drainage structure of tunnel transverse section

**Figure 9:**
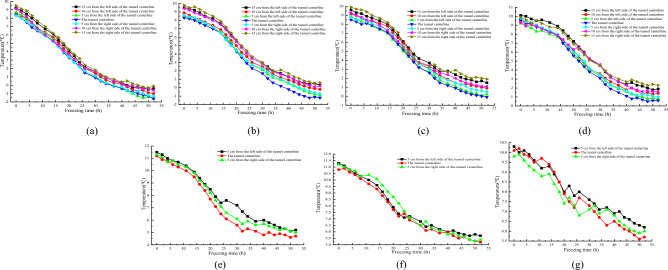
Relationship between transverse sectional temperature and freezing time at the tunnel portal location. (**a**) 5/13 x; (**b**) 10/13 x; (**c**) 16/13 x; (**d**) 21/13 x; (**e**) 28/13 x; (**f**) 33/13 x; (**g**) 38/13 x.

Figure 9 illustrates the variation curve of surrounding rock temperature with freezing time at different positions on the transverse section at a distance of 1/60 from the tunnel opening outside the tunnel.

As shown in Fig. 9, at a depth of 5/13 times the depth of the central drainage ditch, the temperature rapidly decreases with the increase of freezing time at a rate of approximately 0.2–0.3 °C/h after the start of the freezing process. When the freezing time reaches 25 h, the temperature change curve slightly flattens, and the temperature change rate decreases to 0.15 °C/h. However, as the freezing time continues to increase, the temperature still decreases, albeit the temperature drop amplitude decreases. When the freezing time ranges between 40 and 52 h, the amplitude of temperature change further decreases with the increase of freezing time, and the temperature change rate remains around 0.05–0.1 °C/h. When the exchange rate of cold and hot air reaches an equilibrium state, the temperature no longer changes with the continued increase of freezing time, and finally stabilizes. At the depths of 10/13–38/13 times the burial depth of the central drainage ditch, there is a hysteresis phenomenon in the temperature change with freezing time. At the beginning of freezing, the temperature change is relatively small, and the temperature change curve with freezing time is relatively flat, with a small temperature drop rate of roughly 0.05–0.1 °C/h. The temperature freezing time curve undergoes significant changes at the lag time point, and the magnitude and rate of temperature decrease rapidly increase. The temperature freezing time change curve can be fitted with a cubic polynomial through data fitting, and the fitting variance is above 0.99, indicating a high degree of fit. The temperature hysteresis time at each position is shown in Table 4.

**Table 4:** Temperature difference delay time at different buried depths.

Depth	(5/13) x	(10/13) x	(16/13) x	(21/13) x	(28/13) x	(33/13) x	(38/13) x
Latency/h	0	11	14	18	18	16	16

Note: (a) x represents a multiple of the distance from the bottom of the inverted arch.

From Table 4, it can be seen that the overall hysteresis time of temperature change at different burial depths of the tunnel is as follows: as the burial depth of the surrounding rock increases, the hysteresis time of temperature change gradually increases, and the hysteresis phenomenon becomes more obvious. The hysteresis time is directly proportional to the burial depth of the surrounding rock^22^.

**Figure 10:**
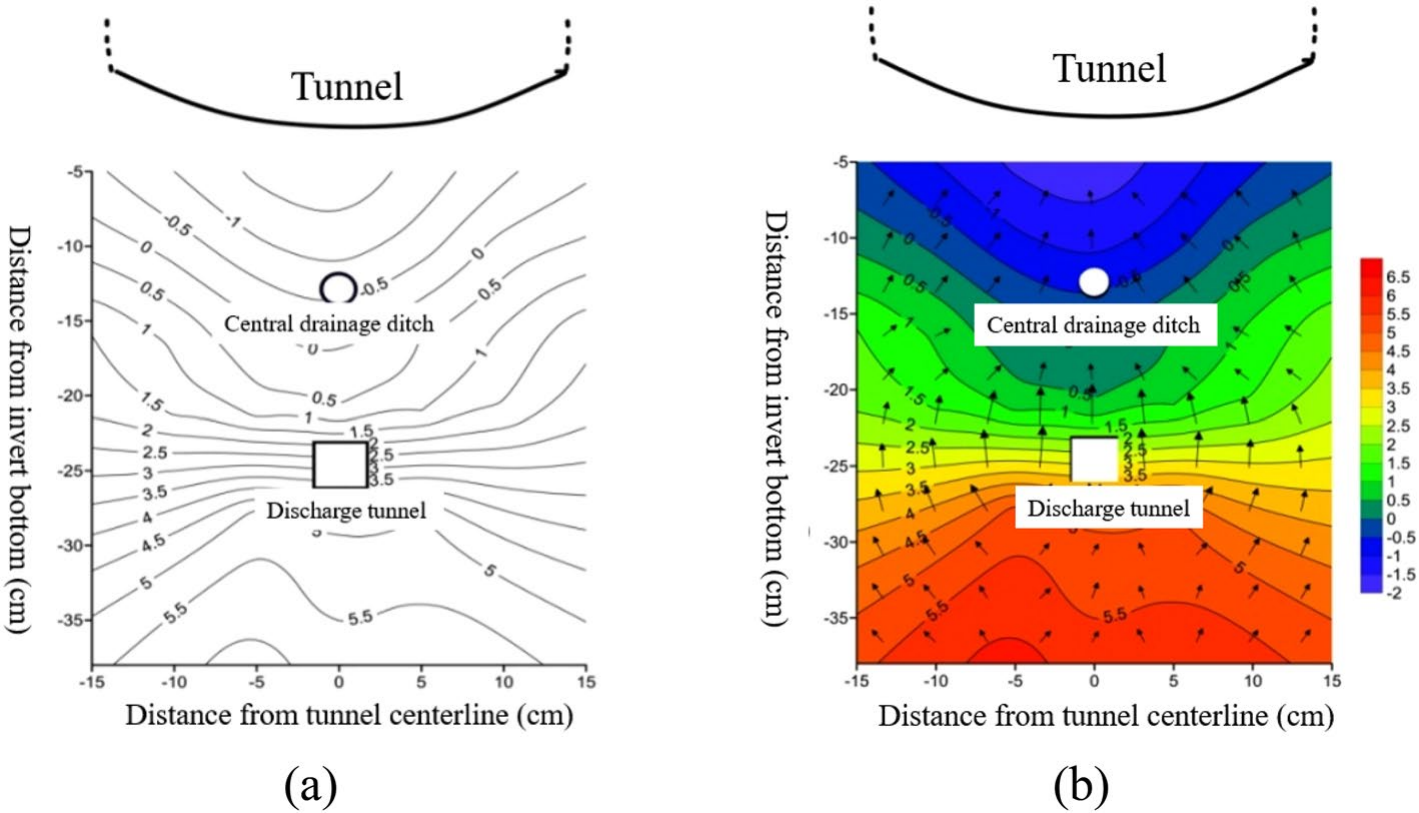
Temperature changes at different locations on the tunnel transverse section. (**a**) Temperature contour map of tunnel transverse; (**b**) temperature vector map of tunnel transverse.

Figure 10 shows the variation of surrounding rock temperature at different positions on the transverse section at a distance of 1/60 from the tunnel opening outside the tunnel.

From Fig. 10, it is evident that at the same burial depth, the temperature at the centerline of the tunnel presents as the lowest within a range of 15 cm from the left to the right sides of the centerline. As the distance from the centerline increases, the temperature gradually increases, and the maximum temperature difference between the centerline of the tunnel and the positions on the left and right sides notes at 2.0 °C. From below the inverted arch of the tunnel, as the burial depth of the temperature sensor increases, the temperature value gradually increases. The influence of atmospheric temperature changes on the temperature sensor decreases, and the impact on the temperature field of the surrounding rock in the lower part of the tunnel also gradually decreases. Shallow buried temperature sensors are more sensitive to external atmospheric changes and are affected greatly by external temperatures.

A negative temperature distribution is observed within the range of 16/13 times the burial depth of the central drainage ditch to the inverted arch, while a negative temperature distribution area is noted around the central drainage ditch with a temperature range of about − 0.3 to − 0.8 °C and a minimum temperature of − 0.8 °C. The temperature distribution around the anti-cold drainage tunnel shows a positive distribution, with a temperature variation range of 1.9–3.5 °C, a minimum temperature of 1.9 °C and a maximum temperature difference of approximately 1.4 °C. The minimum temperature value at a depth of 5/13 times the central drainage ditch is − 1.8 °C, the minimum temperature at a depth of 10/13 times the central drainage ditch is − 1.2 °C, and the minimum temperature at a depth of 38/13 times the central drainage ditch is 5.7 °C. As the burial depth increases, the minimum temperature value in the surrounding rock beneath the tunnel gradually increases, and the temperature progressively becomes positive. The greater the burial depth, the closer the temperature approaches a positive value.

In order to better express the relationship between temperature and freezing time, a cubic function was used to fit the curve, and the fitting results are shown in Table 5.

**Table 5:** Parameters of data fitting equation.

Parameter	Place						
	(5/13) x	(10/13) x	(16/13) x	(21/13) x	(28/13) x	(33/13) x	(38/13) x
C	8.75055	8.96239	8.7354	8.71515	10.54251	10.44898	10.55038
B1	− 0.27113	− 0.25218	− 0.1772	− 0.09119	− 0.05502	− 0.01591	− 0.02026
B2	− 0.00382	− 0.00322	− 0.0038	− 0.00349	− 0.00486	− 0.00622	− 0.00692
B3	9.414E−5	8.22E−5	7.612E−5	5.77E−5	7.46E−5	8.67E−5	1.02E−4
R2	0.99387	0.99004	0.99338	0.99082	0.99098	0.99116	0.99047

Note: (a) x represents a multiple of the distance from the bottom of the inverted arch.

According to the parameters of the fitting equation in the Table 5, the variance of the curve fitting equation at different burial depths is greater than 0.99, indicating that fitting the temperature freezing time change curve with a cubic function is reasonable.

In summary, under the insulation conditions of the inverted arch, central drainage ditch, and anti-cold drainage tunnel, there is a hysteresis phenomenon in the temperature change curve with freezing time, which is manifested as the temperature hysteresis time gradually increases with the increase of burial depth, and the temperature hysteresis phenomenon becomes more obvious. The experimental results show that only at 5/13 times the burial depth of the central drainage ditch, the temperature hysteresis time is 0. After the freezing begins, the temperature rapidly decreases with the increase of freezing time. The law of change can be represented by a cubic polynomial. At the outer section of the tunnel, the temperature on both sides of the tunnel centerline increases with the increase of distance from the centerline, and the temperature shows a “v” shape.

#### Temperature distribution characteristics around the tunnel axial drainage structure

**Figure 11:**
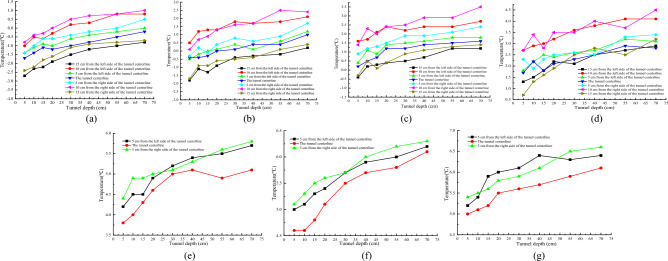
Relationship between transverse sectional temperature and freezing time at the tunnel portal location. (**a**) 5/13 x; (**b**) 10/13 x; (**c**) 16/13 x; (**d**) 21/13 x; (**e**) 28/13 x; (**f**) 33/13 x; (**g**) 38/13 x.

Figure 11a–g respectively show the temperature variation curves of different burial depths in the tunnel with increasing depth.

From Fig. 11, it can be seen that the temperature is the lowest at the position 1/60 of the tunnel depth. As the tunnel depth decreases, the temperature values at each position gradually decrease, and the overall temperature change shows that the temperature value gradually increases as the tunnel depth increases. The influence of cold air on various sensors at the depth of 1/60 of the tunnel entrance is relatively large, and the exchange of cold and hot air is also relatively large. Therefore, the temperature at the tunnel entrance is relatively low. The temperature of the surrounding rock in the lower part of the tunnel gradually increases with the increase of tunnel depth. The temperature changes significantly with the depth of the tunnel from 1/60 to 2/15 of the tunnel depth. As the tunnel depth increases, the amplitude of temperature rise gradually decreases. When the tunnel depth is within the range of 2/15–7/30 of the tunnel depth, the amplitude of temperature rise significantly decreases, and the overall change is relatively gentle. The maximum temperature difference between the measurement points at the same horizontal position as the tunnel depth of 1/60 and the tunnel depth of 7/30 is within the range of 1.4–1.9 °C.

**Figure 12:**
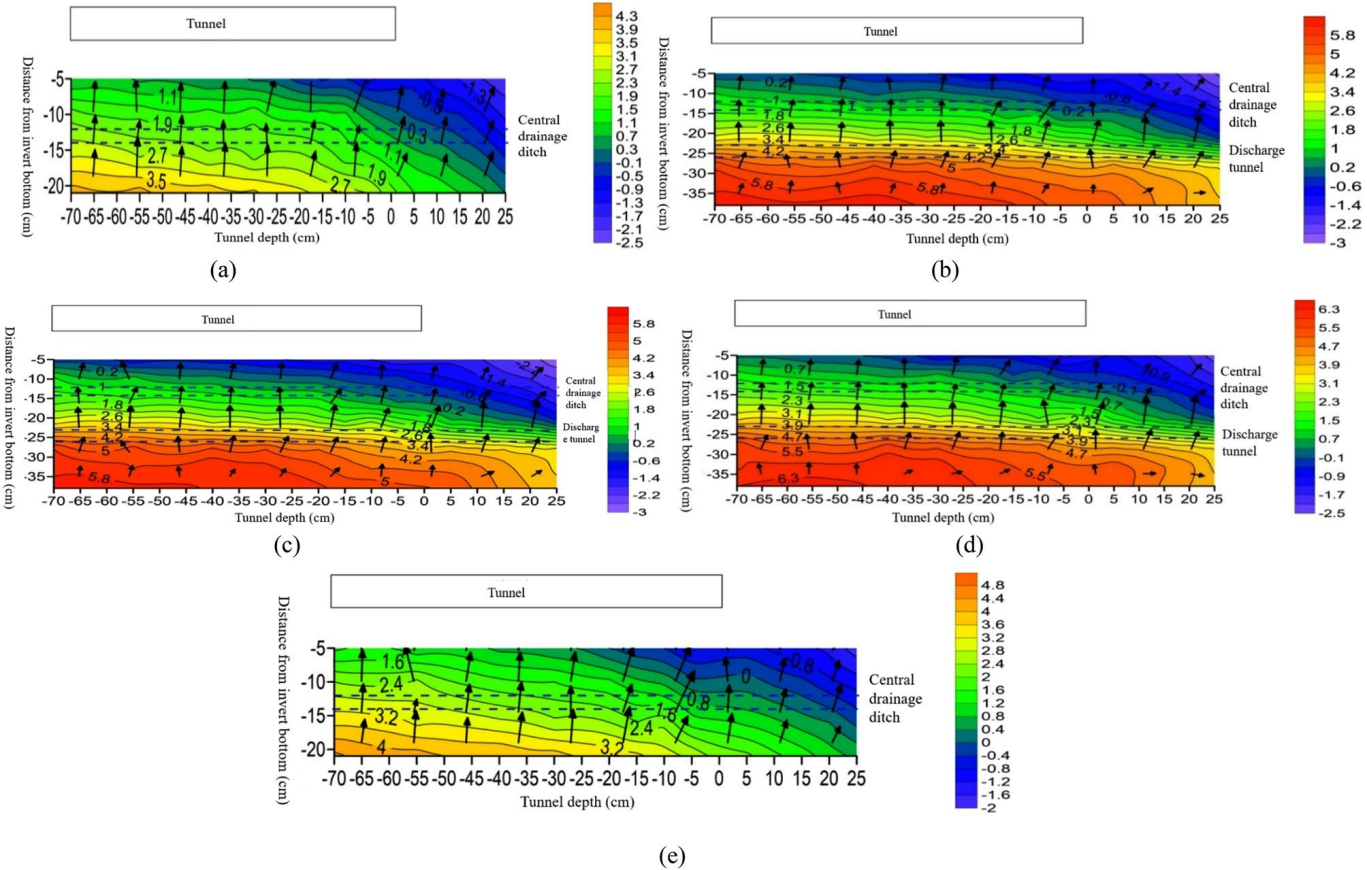
Vector diagram of longitudinal temperature changes at different locations of the tunnel. (**a**) Temperature variation vector map at 10 cm on the left side of the tunnel centerline; (**b**) temperature variation vector map at 5 cm on the left side of the tunnel centerline; (**c**) vector map of temperature changes at the centerline of the tunnel; (**d**) temperature variation vector map at 5 cm on the right side of the tunnel centerline; (**e**) temperature variation vector map at 10 cm on the right side of the tunnel centerline.

From Fig. 12a and e, it is evident that the temperature changes along the axial direction at 10 cm positions on both sides of the tunnel centerline are roughly the same. From 7/30 tunnel depth to the position near the tunnel entrance, the temperature values at different burial depths below the inverted arch decrease with decreasing tunnel depth, and the temperature value is the lowest at 1/12 tunnel depth outside the tunnel entrance. Negative temperatures begin to appear around the central drainage ditch outside the tunnel, while positive temperatures occur within the depth range of the tunnel. From Fig. 12b–d, the temperature variation along the axial direction at the center line of the tunnel and its 5 cm positions on both sides is roughly as follows: from 7/30 tunnel depth to 1/12 tunnel depth outside the tunnel, the temperature decreases with decreasing tunnel depth. The highest temperature at the same burial depth occurs at 7/30 tunnel depth, and the lowest temperature occurs at 1/12 tunnel depth outside the tunnel. There is a negative temperature distribution around the central drainage ditch at 5 cm on both sides of the tunnel centerline, but slight differences occur in the negative temperature distribution areas. There is no negative temperature around the anti-cold drainage tunnel, and all measuring points show positive temperatures. The temperature variation pattern shows that as the depth decreases from inside the tunnel to outside, the temperature gradually decreases.

In summary, after insulation of the inverted arch, central drainage ditch, and anti-cold drainage tunnel, the overall temperature axial variation pattern at different burial depths is as follows: as the tunnel depth increases, the temperature of the surrounding rock in the lower part of the tunnel gradually increases, and the temperature change amplitude gradually decreases with increasing depth. After insulation is installed, the central drainage ditch only experiences temperatures less than 0 °C within a certain range outside the tunnel, while the temperature around the anti-cold drainage tunnel remains above 0 °C throughout the entire experimental section, and there is no freezing phenomenon.

## Conclusions

The article analyzes the temperature field distribution characteristics of tunnel surrounding rock under different insulation layer layout methods for tunnel drainage structure insulation layer, and the conclusions are as follows:

In the early stage of freezing, shallow surrounding rock temperature decreases rapidly with the increase of freezing time. As the burial depth increases, there is a significant lag phenomenon in the temperature change at each measurement point. The lag time of temperature change gradually increases. Negative temperatures still occur within a certain range around the central drainage ditch. Cubic polynomial of temperature variation curve with freezing time. At the longitudinal section, as the tunnel depth increases, the temperature of the surrounding rock at the lower part of the tunnel gradually increases, and the temperature change amplitude gradually decreases with the increase of depth.

At the same burial depth, the temperature near the centerline is relatively low. As the distance from the centerline increases, the temperature value gradually increases; the temperature variation pattern at the inside the tunnel is different from that at the outer section. The temperature at the center line of the tunnel is lower than within a range of 10 cm on both sides, but the temperature is the lowest at 15 cm on both sides of the center line.

When both the anti cold drainage tunnel and the central drainage ditch are equipped with insulation layers, negative temperature only appears within a certain range outside the tunnel at the central drainage ditch. However, when the insulation layer is only installed in the central drainage ditch, significant negative temperature appears around the central drainage ditch. Due to the distance between the cold water drainage tunnel and the surrounding rock, the two layout methods have less effect on the cold water drainage tunnel. In practical engineering, full consideration should be given to the burial depth of the central drainage ditch and the anti cold drainage tunnel, and insulation layer treatment should be applied to both.

## Data Availability

All data generated or analysed during this study are included in this published article.
